# The role of nitric oxide in pre-synaptic plasticity and homeostasis

**DOI:** 10.3389/fncel.2013.00190

**Published:** 2013-10-31

**Authors:** Neil Hardingham, James Dachtler, Kevin Fox

**Affiliations:** School of Biosciences, Cardiff UniversityCardiff, UK

**Keywords:** LTP (Long Term Potentiation), synaptic plasticity, NOS1, experience-dependent plasticity, guanylate cyclase

## Abstract

Since the observation that nitric oxide (NO) can act as an intercellular messenger in the brain, the past 25 years have witnessed the steady accumulation of evidence that it acts pre-synaptically at both glutamatergic and GABAergic synapses to alter release-probability in synaptic plasticity. NO does so by acting on the synaptic machinery involved in transmitter release and, in a coordinated fashion, on vesicular recycling mechanisms. In this review, we examine the body of evidence for NO acting as a retrograde factor at synapses, and the evidence from *in vivo* and *in vitro* studies that specifically establish NOS1 (neuronal nitric oxide synthase) as the important isoform of NO synthase in this process. The NOS1 isoform is found at two very different locations and at two different spatial scales both in the cortex and hippocampus. On the one hand it is located diffusely in the cytoplasm of a small population of GABAergic neurons and on the other hand the alpha isoform is located discretely at the post-synaptic density (PSD) in spines of pyramidal cells. The present evidence is that the number of NOS1 molecules that exist at the PSD are so low that a spine can only give rise to modest concentrations of NO and therefore only exert a very local action. The NO receptor guanylate cyclase is located both pre- and post-synaptically and this suggests a role for NO in the coordination of local pre- and post-synaptic function during plasticity at individual synapses. Recent evidence shows that NOS1 is also located post-synaptic to GABAergic synapses and plays a pre-synaptic role in GABAergic plasticity as well as glutamatergic plasticity. Studies on the function of NO in plasticity at the cellular level are corroborated by evidence that NO is also involved in experience-dependent plasticity in the cerebral cortex.

## Introduction

Nitric oxide is a ubiquitous signaling molecule in the brain and in other organs of the body. It is involved in an almost bewildering array of functions. Consequently, there have been many reviews over the years that have described its role in retrograde signaling (Brenman and Bredt, 1997), cellular function (Garthwaite, 2008), synaptic plasticity (Holscher, 1997), development (Contestabile, 2000), excitotoxicity (Calabrese et al., 2007), blood flow (Gordon et al., 2007) and mental health (Steinert et al., 2010). However, in this review we focus on the role of NO in synaptic plasticity and specifically its function as a retrograde messenger. It seems fitting to look at the evidence now as it is 25 years since the original discovery that NO (or endothelial derived relaxing factor) might act as an intercellular messenger in the brain (Garthwaite et al., 1988), during which time there has been a steady accumulation of evidence for the role of NO synthase in synaptic plasticity and homeostasis at both excitatory and inhibitory synapses. In the following sections we briefly review the main pathways by which NO acts and the distance over which it acts, before discussing the evidence for its role in synaptic signaling during plasticity and homeostasis.

## Molecular pathways for the action of NO

Nitric oxide is generated by the enzyme NO synthase (NOS). NOS1 (nNOS or neuronal NOS) is one of three major isoforms of NO synthase, the others being NOS2 (iNOS or inducible NOS) and NOS3 (eNOS or endothelial NOS). Many cell types in the body can express NOS2, including immune response cells (Hickey, 2001), glial cells (Nomura and Kitamura, 1993) and neurons (Corsani et al., 2008). Unlike NOS1 and NOS3 that are expressed constitutively, NOS2 is induced by inflammatory cytokines (Saha and Pahan, 2006). Calcium/calmodulin has such a high affinity for NOS2 that its activity is not modulated by this route, which means that NOS2 activity is under the control of cytokines rather than calcium signaling. Antagonists of NOS2 have been reported to reduce synaptic plasticity and alter both spontaneous and evoked synaptic activity in the cortex (Buskila and Amitai, 2010), although NOS1 may also have been affected at the drug concentrations used in this study.

NOS3 was originally isolated from endothelial cells, and along with other NOS isoforms is present in the tissues of the cardiovascular system (Buchwalow et al., 2002). While early reports suggested NOS3 was located in neurons (Dinerman et al., 1994), these findings were later rebutted by the same group (Blackshaw et al., 2003). NOS1 knockouts show that NOS1 is the source of 95% of the NO in the cortex (Huang et al., 1993) and plays a major role in synaptic plasticity (see Section NO Controls Pre-Synaptic Function and The Role of NO in Plasticity). However, tonic levels of NO produced by NOS3 may also play a role in the induction of plasticity (Hopper and Garthwaite, 2006).

### Soluble guanylate cyclase

Soluble guanylyl cyclase (sGC) is the most sensitive receptor for NO, with an EC_50_ in the low nanomolar (nM) range (Roy et al., 2008). A good deal of evidence has been gathered in recent years for its importance in mediating the actions of endogenous NO, predominantly at pre-synaptic locations (Garthwaite, 2010; Neitz et al., 2011; Eguchi et al., 2012; Bartus et al., 2013).

Soluble guanylyl cyclase mediates the production of cGMP from GTP. Three subunits of the protein have been identified, α_1_, α_2_, and β_1_. A functional receptor is a heterodimer consisting of one α and one β subunit. Two isoforms of the receptor exist (α_1_β_1_ and α_2_β_1_) with a complex regional expression. For example, the α_1_β_1_ heteromer is dominant in the caudate-putamen and nucleus accumbens whilst α_2_β_1_ is dominant in the hippocampus and olfactory bulb (Gibb and Garthwaite, 2001; Mergia et al., 2003). The α_2_β_1_ receptor is present at the highest levels in the brain and the α_2_ subunit has been shown to bind to the cell membrane through PSD95 (Russwurm et al., 2001; Mergia et al., 2003), which suggests a post-synaptic localization. The α_2_β_1_ isoform can substitute for most functions of the more widely expressed α_1_β_1_ isoform despite there being a 90% reduction in sGC in the α_1_ KOs (Friebe and Koesling, 2009). However, deletion of the β_1_ subunit eliminates expression of any sGC resulting in an 80% infant mortality within 2 days of birth (Friebe and Koesling, 2009). To date, the two α subunit isoforms have only been found to have distinct functions in the induction of LTP in the visual cortex where both isoforms are necessary (Haghikia et al., 2007).

The guanylyl cyclase receptor consists of a haem group of the type that binds O_2_ in hemoglobin, but when associated with the receptor protein, it exhibits a substantial preference for NO, allowing detection of NO in the presence of at least 10,000 fold excess of O_2_, despite the molecular similarity of the two ligands (Martin et al., 2006).

**Figure 1:**
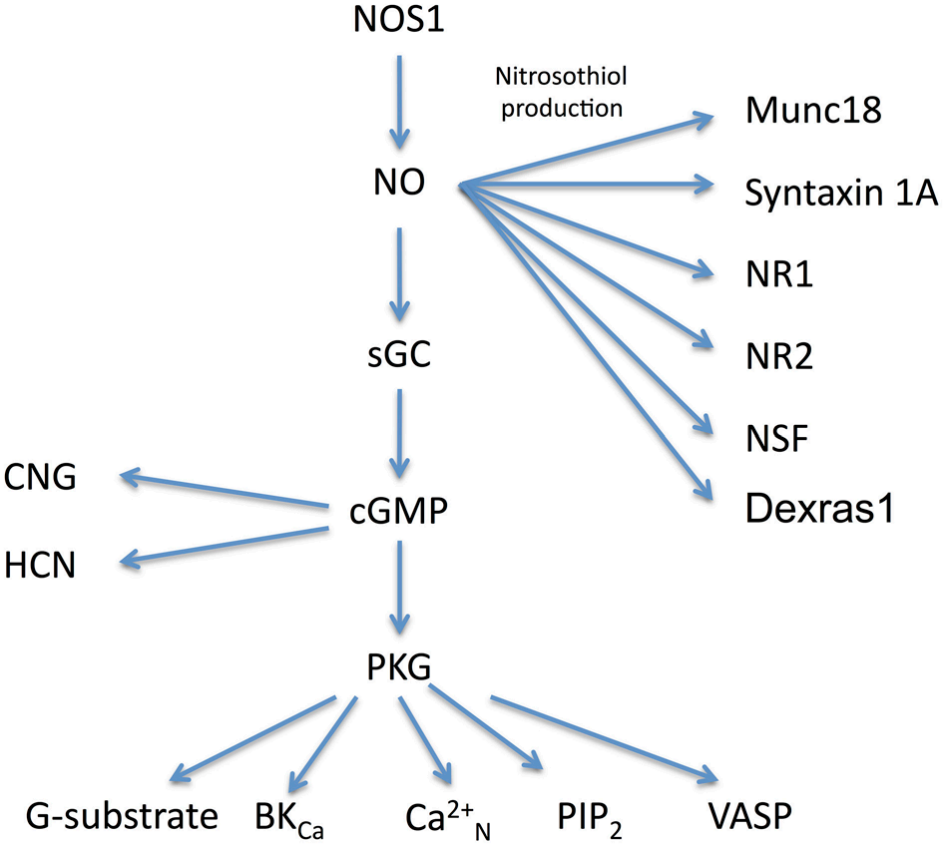
**Molecular signaling pathways for NO.** The main signaling pathways described in the text for NOS1 are shown together with their effector molecules. NO has three main routes of action via nitrosothiol production, cGMP and PKG. Abbreviations: NOS1, Nitric Oxide Synthase 1; NO, Nitric Oxide; sGC, soluble guanylate cyclase; PKG, protein kinase G; G-substrate, a phosphatase inhibitor; BK_*Ca*_, large calcium sensitive potassium channel; Ca2+N, N-type calcium channel; PIP2, phosphotidylinositol 4,5 biphosphate; VASP, vasodilator stimulated phosphoprotien; CNG, cyclic nucleotide gated channel; HCN, hyperpolarization-activated, cyclic nucleotide-modulated channel; Munc18, also known as Sec-1, is a pre-synaptic SNARE associated protein; syntaxin 1A, part of the SNARE complex; NR1, NMDA receptor subunit 1; NR2, NMDA receptor subunit 2; NSF, N-ethylmaleimide sensitive fusion protein. There is evidence for nitrosothiol production in NSF, NR1, and NR2 *in vivo*, but endogenous production of nitrosothiol groups in syntaxin requires confirmation.

The mechanism of activation of sGC by NO is complex and involves a conformational change via binding at the haem site, which enables increased conversion of GTP to cGMP (Roy et al., 2008). NO activates guanylyl cyclase within 20 ms and, following removal of NO, activity decays with a half life of 200 ms (Bellamy and Garthwaite, 2001). With formation of cGMP, a bifurcation occurs in the route of action (Figure 1); one route is for cGMP to affect cGMP-activated protein kinases (cGKs or PKGs). Multiple substrates for PKG have been identified including PKG activated phosphatases, leading indirectly to altered levels of phosphorylation of effector proteins (Schlossmann and Hofmann, 2005). The second major route of action for cGMP is to bind to agonist or regulatory sites on cyclic nucleotide-gated (CNG) ion channels or hyperpolarization-activated, cyclic nucleotide-modulated (HCN) channels.

### Production of nitrosothiol groups

There are a number of cases where NO signaling in the brain is transduced in a cGMP independent manner. The thiol side chains of cysteine residues in proteins can be modified by the addition of an NO group and this outcome could occur by two known routes: the thiol group can be oxidized to a thyl followed by addition of NO, which is known as oxidative nitrosylation, or NO can react with O_2_ to produce N_2_O_3_ which then interacts with the thiol group to produce nitrosothiol, and this process is known as nitrosation (Heinrich et al., 2013). At present, the endogenous route for nitrosothiol production is not known.

A number of pre-synaptic proteins have been identified as potential targets for nitrosothiol production and therefore as a mechanism for mediating alterations in pre-synaptic strength (Figure 1). The t-snare protein synapsin has been identified as a target for nitrosothiol production in pancreatic cells (Wiseman et al., 2011) and syntaxin 1a and n-sec1 (also known as Munc18) have been shown to be a target for nitrosothiol production in neurons (Meffert et al., 1996; Prior and Clague, 2000; Palmer et al., 2008). A small GTPase known as Dexras1 (which can be induced by dexamethasone) is held in close proximity to NOS1 by CAPON (Jaffrey et al., 1998) and can be modified by production of nitrosothiol (Fang et al., 2000).

Nitrosothiol production requires much higher concentrations of NO than activation of sGC and proceeds with slower kinetics. For example, nitrosothiol production in syntaxin 1A occurs with an IC_50_ of 1.1 μM NO (Palmer et al., 2008) compared with the nM range of detection for sGC (Roy et al., 2008). It has been estimated that an NO concentration of 200 μM would require 2 min to produce nitrosothiol groups in half the substrate (Ahern et al., 2002). The high concentrations and slow reaction kinetics of nitrosothiol production raise the question of whether it can occur naturally. Most of the experiments conducted on production of nitrosothiol groups in various proteins use NO donors at quite high levels [for example 100–1000 μM for nitrosothiol production in SNAP25 (Di Stasi et al., 2002)]. However, a technique for detecting nitrosothiol groups in proteins known as the biotin switch method has been used to demonstrate the existence of endogenous nitrosothiol groups *in vivo* by comparing results in wild-type mice with NOS1 knockout mice (Jaffrey et al., 2001). The synaptic proteins that appear to have endogenous nitrosothiol groups using this method include NR1, NR2A (Jaffrey et al., 2001), and NSF (Huang et al., 2005).

It may not be coincidental that some of the molecules shown to have nitrosothiol groups *in vivo* are held in close proximity to NOS1 and thereby experience the higher source concentrations of NO. The NMDA receptor is local to NOS1 by virtue of them both binding to PSD95 and dexras1 is close to NOS1 because both bind to CAPON (Fang et al., 2000). It may also be relevant that nitrosothiol groups occur on molecules that tend to lie close to lipid membranes, in this case synaptic membranes. It has been suggested that the kinetics of the reaction between NO and O_2_ to produce N_2_O_3_ could be increased by NO and O_2_ becoming concentrated in lipid membranes (Heinrich et al., 2013). However, once again it should be emphasized that the endogenous routes for generating nitrosothiol groups on proteins are not known at present.

## The cellular location of NOS1

NOS1 is composed of several splice variants. The long form of NOS is αNOS1 which contains a PDZ binding domain that enables it to bind to the PDZ2 domain of PSD95 (Brenman et al., 1996; Eliasson et al., 1997) localizing NOS1 to the post-synaptic density (see Doucet et al., 2012). There are also shorter splice variants of NOS1 lacking the PDZ domain known as β NOS1 and γNOS1. While the latter is not expressed very highly in the brain, β NOS1 is expressed quite highly in the ventral cochlear nuclei, the striatum and the lateral tegmental nuclei (Eliasson et al., 1997). In the cortex and hippocampus, the current evidence suggests that NOS1 is located in two very different neuronal compartments in two different cell types. On the one hand, NOS1 is located in the cytoplasm of a small subpopulation of GABAergic cells in the cortex and hippocampus and on the other, it is located in a far larger population of excitatory neurons, but highly restricted to the spine head. The ease with which NOS1 can be detected at the two locations depends on the techniques used as described below.

### Light microscopy

**Figure 2:**
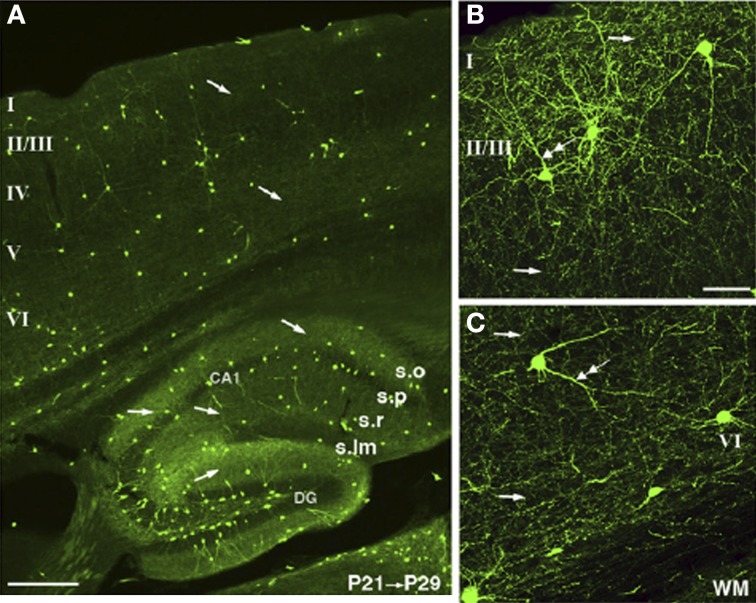
**NOS1 positive cells at the LM level in the Cortex and Hippocampus.** Cells expressing TdTomato fluoresce in nNOS positive cells in an nNOS-CreER;Ai9 mouse. The TdTomato is rendered green in the images. **(A)** The nNOS positive cells make up a small population scattered in cortex and hippocampus. **(B)** A dense and diffuse plexus of neuropil can be seen throughout layer II/III and **(C)** throughout deeper layers of the cortex. Single arrows indicate axons and double arrows dendrites. Adapted from Taniguchi et al. (2011) with kind permission of the authors and Cell press.

The light microscopy (LM) level is sufficient to demonstrate the presence of cytoplasmic NOS1 (Eliasson et al., 1997; Blackshaw et al., 2003; Kubota et al., 2011). LM antibody studies have shown that the strongest NOS1 staining in the neocortex and hippocampus occurs in a small subpopulation of GABAergic neurons (Wendland et al., 1994; Aoki et al., 1997; Blackshaw et al., 2003) that co-express Somatostatin, Neuropeptide Y and the Substance P receptor (Kubota et al., 2011). The NOS1^+^ GABAergic neurons contain both αNOS1 and βNOS1. A significant component of the cytoplasmic staining is attributable to βNOS1 as it persists in αNOS1 knockouts (Eliasson et al., 1997). Weaker labeling of the cortical neuropil is also consistently reported in the same papers. Recent studies using targeted knockin of cre-recombinase into the NOS1 gene and subsequent crosses to GFP reporter lines clearly show two populations of NOS1^+^ GABAergic cells, one of neurogliaform morphology (type II) and the other characterized by long range axonal projections (type I) (Taniguchi et al., 2011). Again the neuropil can be seen throughout the cortical layers including clear axonal labeling (Figure 2). Pyramidal cell labeling is not seen in these cre lines, however, possibly due to the technique only showing high levels of NOS1 expression (Josh Huang personal communication). Weak labeling of CA1 pyramidal cells can be seen using NOS1 antibodies with the right fixative conditions (Burette et al., 2002; Blackshaw et al., 2003) and colocalization of NMDA, PSD95, and NOS1 shows that some of the punctate labeling seen with LM is due to NOS1 in spines (Burette et al., 2002).

### Electron microscopy

**Figure 3:**
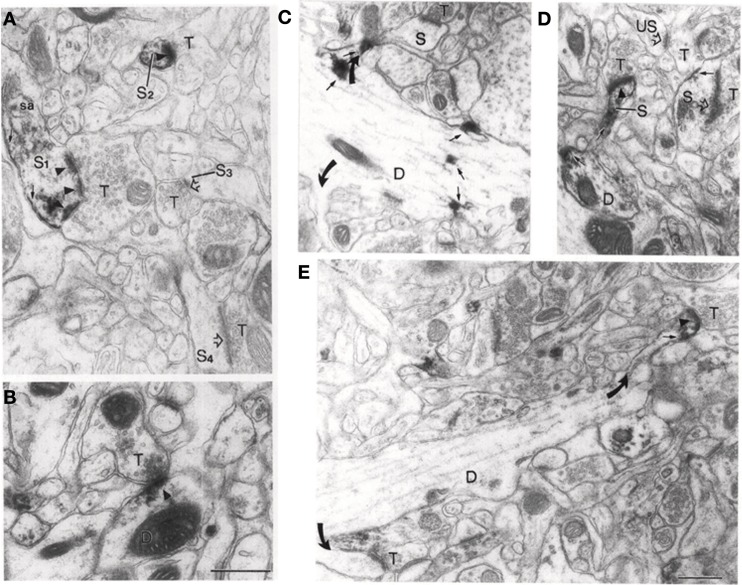
**NOS1 positive spines at the EM level in the Visual Cortex. (A)** Large dendritic spine (S1) with a perforated PSD showing NOS1-immunoreactivity (arrowheads). NOS1 immunoreactivity is also present along the plasma membrane (small arrow) and near the spine apparatus (sa). A second small spine (S2) shows NOS1 immunoreactivity along the plasma membrane and over the PSD. Not all spines are labeled (S3 and S4). T represents unlabeled pre-synaptic terminals. Open arrows mark unlabeled PSDs. **(B)** Axodendritic synapse showing NOS1 labeling of a PSD (arrowhead). **(C)** NOS1 labeling occurs at the spine base (upper curved arrow) and dendritic shaft (small arrows). Lower curved arrow points to an unlabeled spine. S is a spine head and D is a dendritic shaft where limited NOS1 labeling occurs along the plasma membrane. **(D)** NOS1 immunoreactivity over the spine neck (S), plasma membrane forming the spine head (small arrow) and the PSD (filled arrowhead). US marks an unlabeled spine and open arrowheads also mark unlabeled spines and T is the pre-synaptic terminal. **(E)** NOS1 immunoreactivity only in the spine head. Note that in all these cases there is no labeling of the dendritic cytoplasm. Calibration bar = 500 nm. Adapted from Aoki et al. (1998) with kind permission of the author and Elsevier press.

**Figure 4:**
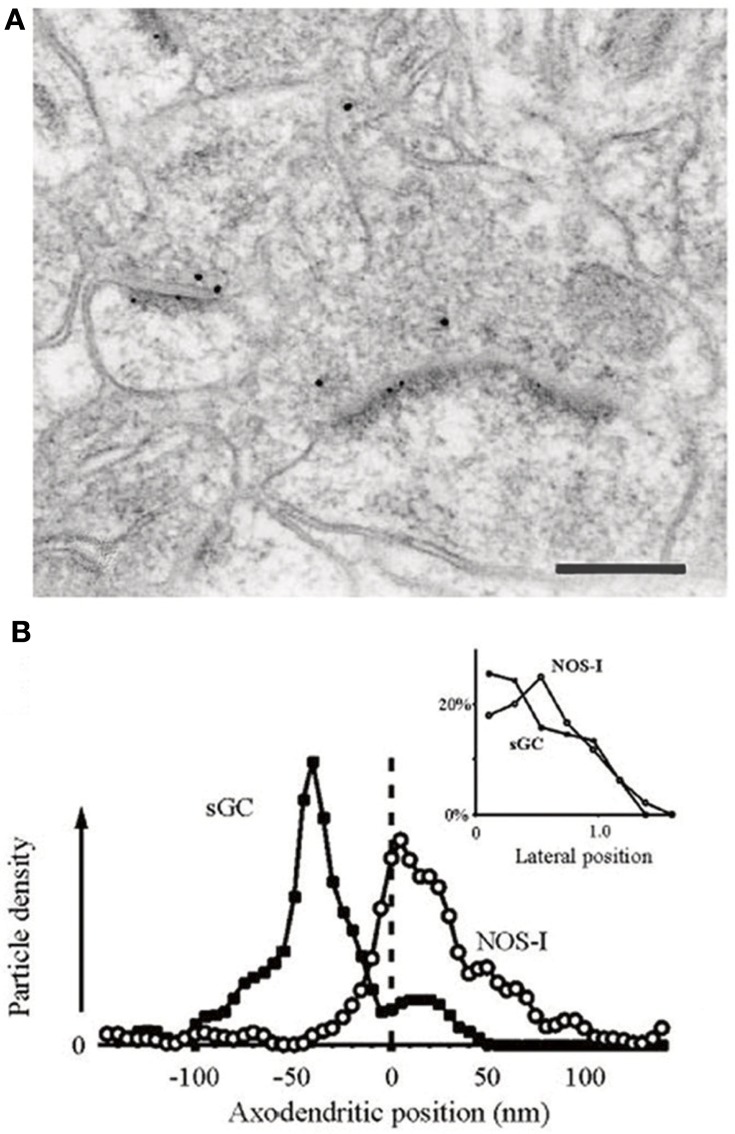
**NOS1 positive spines and sGC positive terminals in the hippocampus. (A)** Positions of gold particles identifying NOSI and sGC located within 150 nm of the post-synaptic membrane. Inset, labeling close to the plasma membrane is concentrated at the synaptic specialization for both antigens. **(B)** Double immunogold labeling showing that NOS1-positive PSDs lie post-synaptic to sGC-positive axon terminals. Small dots are 5 nm gold particles labeling NOS1. Large dots are 10 nm gold particles labeling sGCβ. Scale bar is 200 nm. Adapted from Burette et al. (2002) with kind permission of the authors and the Society for Neuroscience.

Using electron microscopy (EM), much of the neuropil labeling present in LM studies can be seen to reside in the axons of NOS^+^ GABAergic neurons (Aoki et al., 1997). However, EM studies reveal a further component of the neuropil labeling to be due to the very precise and restricted localization of NOS1 in spines, spine heads, and occasionally the plasma membrane of dendrites (Aoki et al., 1997, 1998). The NOS1 visible in the heads of spines in the visual cortex and in some cases at the base of spines accounts for 30–75% of the punctate labeling in cortical electron micrographs (Figure 3). Although the NOS1^+^ GABAergic neurons are sparsely spiny and could theoretically account for some of the NOS1 spine labeling, the extent of the spine labeling seen in EM is too great to be due purely to GABAergic cells (Cheri Aoki personal communication); therefore a considerable amount of spine labeling must be attributable to excitatory pyramidal cells. Furthermore, the NOS1 labeling in spines is quite distinctive in that the labeled spines are joined to dendrites that do not contain NOS1 labeling (Figure 3); if these spines were located on GABAergic cells, the cytoplasm would be labeled as well. EM studies of cortical synapses also show that the gold particle distribution associated with NOS1 labeling is coextensive with that for PSD95 relative to the plasma membrane (Valtschanoff and Weinberg, 2001). Similarly, in the hippocampus, EM studies show that NOS1 is located in dendritic spines on pyramidal cells (Burette et al., 2002). The NO receptor sGC is found pre-synaptic and within 50–150 nm of the NOS (Figure 4). In conclusion, pyramidal cells in the neocortex and hippocampus contain NOS1 that is highly localized to the spine head, spine neck, or plasma membrane of the dendrites and is closely apposed to pre-synaptic sGC.

### Molecular and functional methods

The reason why NOS1 is localized to the spine head is due to the nature of the alpha sub-isoform of NOS1 which contains a PDZ binding domain that enables it to bind to the PDZ2 domain of PSD95 (Brenman et al., 1996; Eliasson et al., 1997). Using proteomic analysis of molecules associated with the NMDA receptor, it has been shown that NOS1 is part of the NMDA signaling complex (Husi et al., 2000). The authors used a combination of immunoaffinity chromatography, immunoprecipitation with an antibody directed against the NR1 subunit, and peptide affinity based on the structure of the NR2B subunit C terminus that binds to the NMDAR-binding protein PSD-95. The structure and binding partners of NOS1 and PSD95 are reviewed in (Zhou and Zhu, 2009) and (Doucet et al., 2012).

Functional assays also demonstrate the synaptic location of NOS1. The functional consequences of disrupting the interaction between NOS1 and PSD95 has been studied by expressing decoy proteins that code for amino acids constituting the PDZ binding domain of αNOS1. For example, glutamate induced activation of p38 normally leads to excitotoxic cell death, but this process can be prevented by expression of the first 300 amino acids of NOS1 (NOS1_1–300_) (Cao et al., 2005). Similarly, cerebral ischemia induced by cerebral artery occlusion leads to cortical damage which can be reduced by NOS1_1–133_ (Zhou et al., 2010) and pTAT-PDZ1-2 (Aarts et al., 2002). Thermal hyperalgesia and chronic mechanical allodynia can be inhibited by intrathecal application of IC8731 or tat-NOS1 (NOS1 1-299) (Florio et al., 2009). These molecules do not act to reduce the enzymatic activity of NOS1, but rather to decrease the coupling between NOS1 and NMDA receptors by disrupting the ability of NOS1 to bind to PSD95 (Florio et al., 2009).

Finally, studies on synaptic plasticity (as described in Section The Role of NO in Plasticity), show that the pre-synaptic NO-dependent component of LTP can be prevented by post-synaptic application of NOS antagonists to layer 2/3 pyramidal cells in the somatosensory cortex (Hardingham and Fox, 2006). Similar results have been demonstrated for layer 5 cortical cells (Sjostrom et al., 2007). This implies that the NO synthase exists in pyramidal cells in the cortex.

In conclusion, LM studies are able to demonstrate the presence of NOS1 in the NOS^+^ GABAergic cells of the neocortex and hippocampus but LM is at the limit for demonstrating its presence in pyramidal cells, while EM, proteomic, and functional analysis are sensitive enough to demonstrate the presence of NOS1 at spines of pyramidal cells.

## The physiological concentration of NO and its distance of action

A theoretical consideration of the rate of production of NO at an individual synapse suggests that NO has a source concentration in the low nanomolar range. Working forward from a knowledge of the rate of NO production per NOS molecule *in vitro* of 20 per second (Santolini et al., 2001) and using an estimate of the number of NMDA receptors and therefore NOS molecules present at a single post-synaptic density, the concentration in the immediate vicinity of the NOS molecule can be estimated at 2.5 nM, falling 10 fold within approximately 700 nm (Hall and Garthwaite, 2009). Working backwards from a measure of NO concentration generated in a cerebellar slice stimulated with NMDA gives a similar rate of production of NO per NOS molecule (10 per second) and a source concentration at the synapse of approximately 0.01–0.1 nM (Wood et al., 2011). A number of studies have reported that NO is produced in the brain in the picomolar range (Wakatsuki et al., 1998; Sato et al., 2006; Wood et al., 2011) and several other labs in the low nM range (<10 nM) (Shibuki and Kimura, 1997; Kimura et al., 1998; Wu et al., 2001, 2002; Sammut et al., 2006, 2007a,b; Ondracek et al., 2008; Sammut and West, 2008).

If the concentration of NO produced at a synapse is in the pM to low nM range, then the rate of inactivation of NO with distance in the brain implies that it can only act over a relatively short range. The most sensitive target for NO is soluble guanylate cyclase (sGC), which can respond to as little as 1 pM NO (Batchelor et al., 2010). The EC_50_ of sGC to NO is thought to be in the low nanomolar range at 1.7 nM (Griffiths et al., 2003). Physiological concentrations of ATP (1 mM) and GTP (0.1 mM), which antagonistically decrease and increase the sensitivity of sGC to NO, respectively, elevate the EC_50_ to 3.4 nM (Roy et al., 2008). Taking into account both the likely concentration of NO at the synapses and the sensitivity of sGC suggests that NO is only likely to act over distances of less than 1 micron.

The lower estimate of NO evolution in the picomolar range would sit on the non-linear cusp of the NO/sGC binding curve (Roy et al., 2008). This raises the interesting possibility that a tonic level of NO production could interact with the NMDA receptor activated NO concentration to boost its effect on sGC. For example, a tonic level of 250 pM NO would move the operating point of the synapse onto the linear part of the NO/sGC curve [see Figure 7B of Roy et al. (2008)]. There is evidence for a tonic level of NO production in the brain originating from both NOS3 and NOS1 (Hopper and Garthwaite, 2006; Dachtler et al., 2011). Furthermore, tonic levels of NO have been found to influence the magnitude of LTP, giving further credence to this notion. NO donors can be shown to facilitate both post-synaptic potentials and LTP (Bohme et al., 1991; Malen and Chapman, 1997; Hardingham and Fox, 2006). The higher estimate of NO release in the nM range would not require background levels of NO to move sGC on to the linear part of its response curve. With either mode of action, NO would only be able to act over a distance of less than about 1 micron, effectively making it a synapse specific signal.

The view of NO as a synapse specific signal does not fit with the notion of NO as a volume transmitter. Nevertheless, there is evidence for NO acting as a volume transmitter in the Calyx of Held (Steinert et al., 2008). Theoretically, all that would be required for higher concentrations of NO would be higher concentrations of the enzyme NOS. It is conceivable that the GABAergic inhibitory cells that express NOS1 at much higher levels than excitatory cells (Figure 2) throughout their cytoplasm could provide such a source. The NOS1^+^ GABAergic cells produce a plexus of fine NOS positive fibers that ramify throughout the cortex and hippocampus, which could aid spatial summation of NO levels. However, little is known of NO release from this small subpopulation of cells at present.

## NO controls pre-synaptic function

The past two decades have seen a steady but decisive accumulation of evidence showing not only that NO acts pre-synaptically on neurotransmitter release, but how it does so (Feil and Kleppisch, 2008). Table 1 is a compilation of papers showing some of the evidence for NO's pre-synaptic action, its retrograde route from post- to pre-synaptic site and its pre-synaptic action in plasticity.

**Table 1:** **Evidence that nitric oxide influences presynaptic function**.

**References**	**Title**	**Presynaptic action?**	**Retrograde messenger?**	**Effect on plasticity?**	**Transmitter**	**Structure (preparation)**
Arancio et al., 1996a	Nitric oxide acts directly in the presynaptic neuron to produce long-term potentiation in cultured hippocampal neurons	✓	✓	✓	Glutamate	Hippocampus (cell culture)
Lange et al., 2012	Heterosynaptic long-term potentiation at interneuron-principal neuron synapses in the amygdala requires nitric oxide signaling	✓	✓	✓	GABA	Amygdala (slices)
O'Dell et al., 1991	Tests of the roles of two diffusible substances in long-term potentiation: evidence for nitric oxide as a possible early retrograde messenger	✓	✓	✓	Glutamate	Hippocampus (slices)
Sjostrom et al., 2007	Multiple forms of long-term plasticity at unitary neocortical layer 5 synapses	✓	✓	✓	Glutamate	Visual cortex (slices)
Hardingham and Fox, 2006	The role of nitric oxide and GluR1 in presynaptic and postsynaptic components of neocortical potentiation	✓	✓	✓	Glutamate	Barrel cortex (slices)
Schuman and Madison, 1991	A requirement for the intercellular messenger nitric oxide in long-term potentiation	✓	✓	✓	Glutamate	Hippocampus (slices)
Volgushev et al., 2000	Retrograde signaling with nitric oxide at neocortical synapses	✓	✓	✓	Glutamate	Visual cortex (slices)
Montague et al., 1994	Role of NO production in NMDA receptor-mediated neuro-transmitter release in cerebral cortex	✓	✓		Glutamate	Neocortex (synaptosomes)
Micheva et al., 2003	Retrograde regulation of synaptic vesicle endocytosis and recycling	✓	✓		Glutamate	Hippocampus (cell culture)
Eguchi et al., 2012	Maturation of a PKG-dependent retrograde mechanism for exoendocytic coupling of synaptic vesicles	✓	✓		Glutamate	MNTB/Caylx of Held (slices)
Lindskog et al., 2010	Postsynaptic GluA1 enables acute retrograde enhancement of presynaptic function to coordinate adaptation to synaptic inactivity	✓		✓	Glutamate	Hippocampus (cell culture)
Qiu and Knopfel, 2007	An NMDA receptor/nitric oxide cascade in presynaptic parallel fiber-Purkinje neuron long-term potentiation	✓		✓	Glutamate	Cerebellum (slices)
Johnstone and Raymond, 2011	A protein synthesis and nitric oxide-dependent presynaptic enhancement in persistent forms of long-term potentiation	✓		✓	Glutamate	Hippocampus (slices)
Stanton et al., 2005	Imaging LTP of presynaptic release of FM1-43 from the rapidly recycling vesicle pool of Schaffer collateral-CA1 synapses in rat hippocampal slices	✓		✓	Glutamate	Hippocampus (slices)
Wang et al., 2005	Presynaptic and postsynaptic roles of NO, cGK, and RhoA in long-lasting potentiation and aggregation of synaptic proteins	✓		✓	Glutamate	Hippocampus (cell culture)
Arancio et al., 2001	Presynaptic role of cGMP-dependent protein kinase during long-lasting potentiation	✓		✓	Glutamate	Hippocampus (cell culture)
Huang et al., 2003	cGMP/protein kinase G-dependent potentiation of glutamatergic transmission induced by nitric oxide in immature rat rostral ventrolateral medulla neurons *in vitro*	✓			Glutamate	Ventrolateral medulla (slices)
Ratnayaka et al., 2012	Recruitment of resting vesicles into recycling pools supports NMDA receptor-dependent synaptic potentiation in cultured hippocampal neurons	✓			Glutamate	Hippocampus (cell culture)
Neitz et al., 2011	Presynaptic nitric oxide/ cGMP facilitates glutamate release via hyperpolarization-activated cyclic nucleotide-gated channels in the hippocampus	✓			Glutamate	Hippocampus (slices)

Much of the detailed evidence for NO's role in transmitter release comes from studies on the glutamatergic system, but a body of work implicates NO in regulating transmitter release from GABAergic (Kawaguchi et al., 1997; Li et al., 2002; Moreno-Lopez et al., 2002; Wall, 2003; Szabadits et al., 2007; Yang et al., 2007; Bright and Brickley, 2008; Xue et al., 2011; Lange et al., 2012) dopaminergic (West et al., 2002) and noradrenergic synapses (Montague et al., 1994; Kodama and Koyama, 2006).

A number of the studies providing evidence for the retrograde action of NO have come from cell cultures. Cell culture preparations have a number of technical advantages that allow the retrograde action of NO to be demonstrated (Table 1). However, since cells in culture are immature, it raises the question of whether NO acts the same way in more mature cells. Nevertheless, a number of studies made on mature neurons in intact slices of hippocampus (O'Dell et al., 1991; Schuman and Madison, 1991), amygdala (Lange et al., 2012), neocortex (Hardingham and Fox, 2006; Sjostrom et al., 2007), the medial nucleus of the trapezoid body (Steinert et al., 2008; Eguchi et al., 2012), cerebellum (Qiu and Knopfel, 2007), and the ventral lateral medulla (Huang et al., 2003), lead to similar conclusions about the action of NO in mature cells, suggesting that NO retains its retrograde pre-synaptic action into adulthood.

**Figure 5:**
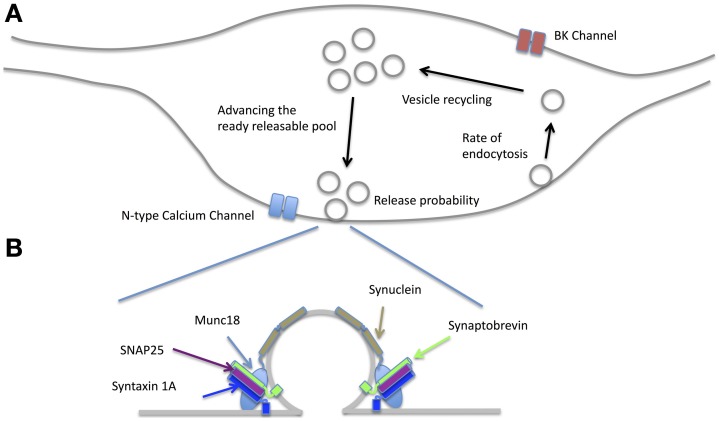
**Effects of NO on the pre-synaptic terminal. (A)** NO affects release-probability most likely through a combination of effects including enhancing N-type calcium channel conductance via PKG, increasing the rate of endocytosis and vesicle recycling as well as altering the balance of the readily releasable pool via PKG and PIP_2_. By acting on BK channels, the probability of action potential failures during moderate spike rates is reduced (see text for the related references). **(B)** There is evidence that many of the SNARE proteins are affected by NO. Syntaxin 1A and SNAP25 can have nitrosothiol groups added although whether this happens at physiological NO concentrations is yet to be established. NO also creates nitrosothiol groups on Munc18 and thereby disinhibits syntaxin from forming the SNARE complex. Alpha Synuclein is also affected by NO signaling. Synaptobrevin is not known to be affected by NO.

In the following sections we briefly review the findings for NO's effects on four aspects of pre-synaptic function; actions at the active zone, on vesicle recycling, effects on the readily releasable pool and actions on pre-synaptic growth. When viewed in combination, these studies suggest that NO may regulate pre-synaptic release by acting in a coordinated and synergistic manner on several aspects of pre-synaptic release (Figure 5).

### Effects on the active zone and transmitter release

Nitric oxide can affect transmitter release by nitrosothiol generation in a number of constituents of the active zone (Figure 5B). For example, nitrosothiol production in syntaxin at Cys(145) has a facilitatory effect on release because it prevents munc18 (also known as n-sec1) from binding to the closed conformation of syntaxin 1a. This allows syntaxin1a to unfold and bind to both VAMP on the vesicle and SNAP25 at the release site, which in turn enables the vesicle to dock to the membrane (Meffert et al., 1996; Palmer et al., 2008). SNAP25 can itself have nitrosothiol groups generated by NO, which may further enhance release (Di Stasi et al., 2002). However, it is not clear at present whether the concentrations of NO necessary for production of nitrosothiol groups are realized at the synapse (see Sections The Cellular Location of NOS1 and The Physiological Concentration of NO and Its Distance of Action).

### Effects on ion channels

Voltage gated ion channels that reside in the pre-synaptic terminal and affect transmitter release have been shown to be NO sensitive. In the peptidergic synapse of the pituitary nerve, NO can increase pre-synaptic release by enhancing the activity of large conductance Ca^2+^ activated K^+^ channels (BK). PKG only activates BK at depolarized potentials, which means that the action potential after-hyperpolarization becomes larger without affecting the spike threshold. Consequently, during prolonged trains of action potentials, the enhanced hyperpolarization provided by BK channels accelerates Na^+^ channel recovery (Klyachko et al., 2001). It can be demonstrated that cytosolic calcium almost doubles in the presence of exogenous cGMP. A possible physiological role for this action is suggested by showing that the action potential success rate during a 25 Hz stimulus train is almost twice as great in the control condition when compared to that in the presence of the NO synthase inhibitor 7-NI or the sGC inhibitor ODQ (Klyachko et al., 2001). In the brainstem, synaptic potentials generated by glutamatergic synapses in the ventrolateral medulla can be enhanced by application of the NOS substrate L-arginine (200 uM) (Huang et al., 2003). This effect can be shown to be due to NO acting via a cGMP/protein kinase G-dependent pathway on N-type calcium channels (Huang et al., 2003). It is not known at present whether BK or N-type calcium channels are affected by NO in the cortex or hippocampus.

One other means by which NO may affect transmitter release in some types of neuron is by stimulating the production of cGMP, which directly gates cyclic nucleotide gated channels (Neitz et al., 2011). Cyclic nucleotide gated (CNG) channels are well known for their function in transmitter release in some classes of cell, for example photoreceptors (Rieke and Schwartz, 1994) and olfactory epithelial cells (Leinders-Zufall et al., 2007). However, the distribution of CNG channels is more widespread and roughly mirrors the distribution of the NO/cGMP system (Kingston et al., 1999). For example, CNG channels are present in the rat hippocampus (Kingston et al., 1999) and may be involved in the induction of theta burst LTP in mouse hippocampus (Parent et al., 1998). While native heteromeric CNG channels formed by alpha and beta subunits are gated by cGMP, homomeric channels comprising just the beta subunit are directly activated by NO (Broillet and Firestein, 1997), raising the possibility that NO might act on native CNG channels by two routes. Finally, at the glutamatergic neuromuscular junction in Drosophila, calcium independent vesicular release can result from cGMP triggered by NO (Wildemann and Bicker, 1999), although the exact downstream processes by which this occurs are not known. Calcium independent vesicular release can also be observed in hippocampal synaptosomes (Meffert et al., 1994).

### Effects on vesicle recycling

In order to sustain synaptic release over a period of time, the rate of vesicle recycling needs to at least equal the rate of vesicle exocytosis. This issue is particularly problematic for synapses that release transmitter at high rates, such as those located at the Calyx of Held that terminate on neurons of the medial nucleus of the trapezoid body (MNTB). Part of the solution to this problem at the Calyx is provided by linking vesicle recycling to retrograde release of post-synaptic NO. Activation of the post synaptic MNTB neurons is related to the level of NO production (Steinert et al., 2008), which then drives the level of pre-synaptic cGMP production and hence the level of PKG activity (Eguchi et al., 2012). Finally, activation of PKG up-regulates PIP2, which increases the rate of endocytosis (Eguchi et al., 2012). This homeostatic mechanism therefore links pre-synaptic rate of release (which is sensed by post-synaptic NOS1) to the rate of pre-synaptic vesicle recycling (Figure 5). Regulation of the recycling rate has also been demonstrated in the hippocampus, where a very similar retrograde NO—pre-synaptic cGMP/PIP2 cascade regulates the rate of endocytosis and recycling (Micheva et al., 2003).

### Effects on availability and size of the readily releasable pool (RRP)

Studies aimed at investigating the nature of synaptic plasticity have shown that LTP is accompanied by an increase (and LTD a decrease) in the rate of vesicular release from the readily releasable pool (RRP). The LTP process is NMDA receptor-, tyrosine kinase- and NO-dependent while the LTD process is NMDA-, NO- and PKG-dependent (Stanton et al., 2003, 2005). Studies have shown that the size of the RRP can be modulated by NO (Figure 5). For example, in the case of LTP, NMDA receptor activation leads to NO and calcineurin activation, which combine to increase the proportion of vesicles available for release (i.e., increase the RRP) (Ratnayaka et al., 2012). Once again this can be seen as a homeostatic response to an increase in release probability brought about by the process of LTP itself. The two processes are coordinated because NO is involved both in increasing transmitter release and increasing the size of the readily releasable pool.

### Effects on growth of pre-synaptic terminals

Nitric oxide also affects the growth and formation of new pre-synaptic terminals and can lead to the formation of multi-innervated spines. Long lasting potentiation leads to an increase in pre- and post-synaptic proteins in hippocampal cell cultures. GluA1 subunits of the AMPA receptor increase post-synaptically and synaptophysin increases pre-synaptically (Antonova et al., 2001). Furthermore, the two synaptic markers co-localize at higher frequency following long lasting potentiation, indicating that new synapses are formed. It has been shown that NMDA receptors, NO and actin are required for the pre-synaptic changes. NO acts via PKG to phosphorylate VASP (which acts on actin) and also via cGMP to act in parallel and downstream of RhoGTPase (Wang et al., 2005).

Further evidence for the role of NO in pre-synaptic growth comes from studies manipulating the PDZ2 domain of PSD95 (which is the PDZ domain that binds NOS1). Up-regulation of PSD95 in cultured hippocampal neurons or treatment with an NO donor leads to the formation of multi-innervated spines (MIS). However, if the PDZ2 domain on PSD95 is deleted, thereby dissociating NOS1 from PSD95, multi-innervated spines fail to form (Nikonenko et al., 2008). Similarly, down regulating NOS1 expression with iRNA also prevents MIS from forming (Nikonenko et al., 2008). Finally, increasing SAP97 expression leads to an increase in PSD95 and again an increase in MIS (Poglia et al., 2011). This effect is blocked by NOS antagonists (Poglia et al., 2011).

In conclusion, the studies cited above show that NO is not only involved in the relatively short term changes involved in transmitter release, such as recycling rates and availability of vesicles, but also, in the long-term, in increasing the availability of transmitter by formation of new pre-synaptic terminals, which results in dendritic spines receiving extra pre-synaptic terminals. Such processes could find application in synaptic plasticity. In the following section we review the function of NO in plasticity and examine to what extent the retrograde route of action is involved.

## The role of NO in plasticity

### NO-dependent pre-synaptic plasticity

Some of the earliest studies on the role of NO in synaptic plasticity indicated that it might act at a pre-synaptic locus (O'Dell et al., 1991). Exogenous NO applied to neurons in a hippocampal slice increased spontaneous mini EPSCs and hemoglobin acting as an extracellular scavenger for NO was found to prevent LTP (O'Dell et al., 1991). Indeed, initial studies on the mechanisms of LTP itself provided evidence for a pre-synaptic locus of LTP expression (Malinow and Tsien, 1990). In a series of experiments on cultured hippocampal neurons, Arancio and colleagues showed that cGMP (the downstream effector of NO) needs to be pre-synaptic and NOS post-synaptic to produce plasticity. First, cGMP causes an increase in EPSC amplitude when injected into the pre-synaptic but not the post-synaptic cell (Arancio et al., 1995). Second, application of a PKG antagonist peptide blocks tetanus induced LTP when injected into the pre-synaptic but not the post-synaptic neuron (Arancio et al., 2001). Third, application of a cGMP analogue increases miniature EPSC frequency and this effect is blocked by a post-synaptically but not pre-synaptically injected NOS inhibitor (Arancio et al., 1996a). Forth, a pre-synaptic injection of an NO scavenger also abolishes LTP (Arancio et al., 1996b). More recent work employing fluorescent markers of pre-synaptic function have visualized the pre-synaptic effect of NO in potentiation. Fluorescence imaging of FM-styryl dyes and synaptophysinI-pHluorin has shown that increases to the pre-synaptic recycling pool fraction following synaptic strengthening are dependent upon both NMDA receptor activation and NO release (Ratnayaka et al., 2012).

### The effect of initial release-probability on the locus of plasticity

Early studies on hippocampal plasticity showed that the initial release-probability of the synapse influences whether a pre- or post-synaptic change occurs following LTP (Larkman et al., 1992). If the release-probability of the synapse is low initially then pre-synaptic plasticity occurs, whereas if the pre-synaptic release-probability is high, then a post-synaptic change occurs (Larkman et al., 1992). A similar principal operates at neocortical synapses. In visual cortex, the initial release-probability of the synapse, as judged by the paired pulse ratio (PPR), is predictive of whether NO-dependent potentiation occurs. Using a purely post-synaptic tetanus (without intentionally eliciting action potentials in the pre-synaptic terminals), potentiation occurs in synapses with a low initial PPR and depression or no change occurs in synapses with a high initial PPR (Volgushev et al., 2000). The same conclusion is arrived at if a paired pre- and post-spike conditioning protocol is used. Low release-probability synapses potentiate via changes in release-probability and high release-probability synapses depress (Hardingham et al., 2007). This normalization process causes the population of connections to adopt a more homogenous set of release probabilities after the protocol. These studies lead to two important conclusions; first, the direction of pre-synaptic plasticity acts in a homeostatic manner to move release-probability to an intermediate value and second, that pre-synaptic plasticity occurs provided that there is sufficient dynamic range for it to occur. There is less scope for increasing release-probability at a high release-probability synapse than at a low release-probability synapse. Potentially, a high release-probability synapse could show pre-synaptic potentiation by growth and/or production of MIS, which can occur and is NO-dependent (section Effects on Growth of Pre-Synaptic Terminals), but structural changes are unlikely within the timescale of an LTP experiment.

Since the initial release-probability of the synapse is an important determinant of the locus of plasticity and in which direction it operates, factors that control initial release-probability will determine the level and form of pre-synaptic plasticity. Adenosine is known to affect release-probability (Prince and Stevens, 1992) and a recent study in layer 5 of the somatosensory cortex has shown that adenosine levels are low early in development (P11-P22) and higher in older animals (P28-32) (Kerr et al., 2013). This maturational change means that adenosine reduces release-probability in older animals, thereby increasing the dynamic range for pre-synaptic potentiation. Some mutant mice strains have unusually low initial release-probability synapses that can provide an increased dynamic range for LTP. For example, H-Ras^G12V^ mice have low release-probability synapses in the visual cortex, as judged by short-term dynamics and mini EPSP frequency, and consequently enhanced LTP with an increased pre-synaptic component (Kaneko et al., 2010).

### Pre- and post-synaptic components of plasticity

Early studies on the role of NO in LTP using NO antagonists often found an absolute requirement for NO (Bohme et al., 1991; O'Dell et al., 1991; Schuman and Madison, 1991; Haley et al., 1993; Doyle et al., 1996; Malen and Chapman, 1997), whereas more recent studies have found LTP to be reduced rather than abolished in the absence of NO, both in the hippocampus (Phillips et al., 2008) and in the neocortex (Hardingham and Fox, 2006).

**Figure 6:**
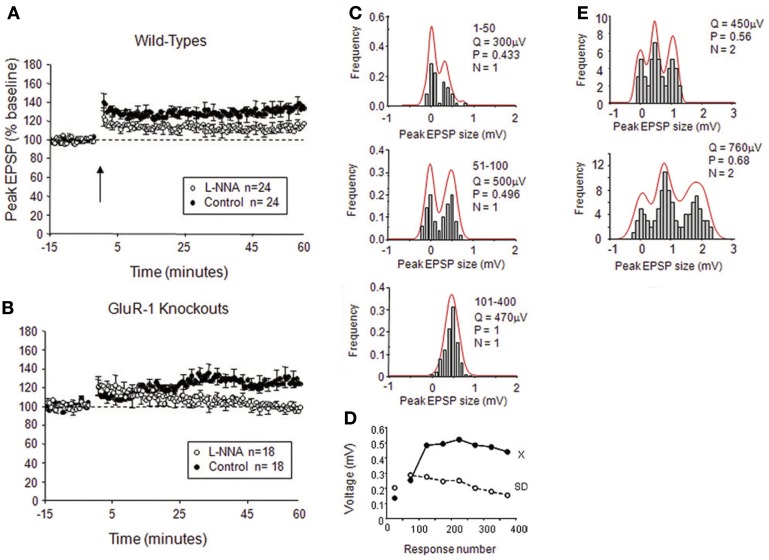
**Effects of NO on release-probability in cortical LTP. (A)** Intracellular application of the NOS antagonist L-NNA reduces but does not abolish spike pairing LTP in wild-type mice. **(B)** Intracellular application of L-NNA abolishes LTP in mice lacking the GluA1 subunit of the AMPA receptor. **(C)** Examples of quantal analysis from a single release site input onto a layer II/III neuron from a wild-type mouse; note that LTP occurs by an increase in release-probability and quantal amplitude. **(D)** The plot of EPSP amplitude and standard deviation for the example in **(C)** during the course of LTP (x = mean, SD = standard deviation). **(E)** Example of quantal analysis from a double release site case in a wild-type treated with intracellular L-NNA; note that LTP occurs largely by an increase in quantal amplitude with a minor increase in release-probability. Q is quantal amplitude, P is release-probability, and N is the number of release sites. Adapted from Hardingham and Fox (2006) with permission of the Society for Neuroscience.

In the neocortex, LTP occurs as a mixture of pre- and post-synaptic changes, but the two components can be dissociated, either by blocking NOS post-synaptically or knocking out GluA1 (Hardingham and Fox, 2006). When a NOS inhibitor is introduced to the post-synaptic neuron via the electrode, plasticity proceeds by changes in quantal amplitude without changes in the variance of the response amplitude (Hardingham and Fox, 2006). Similarly, where single or double quantal release peaks are isolated in layer 2/3 neuones, LTP occurs by changing the quantal amplitude without changes in release-probability (Figure 6). Conversely, in GluA1 knockouts, LTP results in changes in release-probability (*NP*_*r*_) without changes in quantal amplitude (*Q*) (Hardingham and Fox, 2006). Given that potentiation is NO-dependent in GluA1 knockouts this implies that NO acts via a pre-synaptic mechanism in neocortex (Hardingham and Fox, 2006).

The situation is similar in the mature hippocampus, in that the plasticity present in the GluA1 knockouts is largely NO-dependent (Phillips et al., 2008; Romberg et al., 2009), but it is not clear in this case whether the locus of NO-dependent plasticity is pre- or post-synaptic, or perhaps both. Phillips et al. (2008) suggested a pre-synaptic origin for NO-dependent LTP based on the decrease in PPR for 14/21 cases following potentiation, while Romberg et al. (2009) found no change in average PPR. As noted above, it may be that the initial release-probability present at a particular connection affects the likelihood of pre-synaptic plasticity at that synapse (see section The Effect of Initial Release-Probability on the Locus of Plasticity).

Nitric oxide is also known to affect post-synaptic AMPA receptor trafficking; NO increases GluA1 insertion acting via sGC and protein kinase G (PKG) (Serulle et al., 2008) and GluA2 heteromer insertion by production of nitrosothiol groups on NSF (*N*-ethylmaleimide-sensitive factor) (Huang et al., 2005). Furthermore, endogenous NSF does appear to contain nitrosothiol groups *in vivo*. (Huang et al., 2005). However, the GluA1 insertion mechanism cannot be the one operating in the GluA1 knockouts, leaving the GluA2 mechanism as the most likely to be operating in these studies. This view is given further support by data showing the PKC dependence of LTP in the GluA1 knockout animals (Romberg et al., 2009). Nevertheless, in wild-types it is possible that both GluA1 and GluA2 are controlled by NO signaling. Accumulation of cGMP in hippocampal cells has recently been demonstrated using NO donors (Bartus et al., 2013) giving further credence to a post-synaptic role for NO. Furthermore, there is some evidence that dexras1 is activated by NO and is located post-synaptically due to CAPON binding dexras1 and NOS1 (Fang et al., 2000; Cheah et al., 2006). Together with the substantial evidence that NO acts pre-synaptically (Section NO Controls Pre-Synaptic Function), these findings raise the intriguing possibility that NO might play a role in coordinating pre- and post-synaptic changes at excitatory synapses during plasticity.

### Evidence for the role of NO in experience-dependent plasticity

There is an extensive literature on the role of NO in learning and memory. Peripheral administration of NOS inhibitors have been shown to impair spatial memory acquisition or recall (Bohme et al., 1993; Chapman et al., 1992; Zou et al., 1998; Majlessi et al., 2008), social interactions (Bohme et al., 1993) and object recognition memory (Cobb et al., 1995). Central administration of NOS antagonists also alters behavior, including spatial learning in the Morris water maze and the passive avoidance test (Qiang et al., 1997; Majlessi et al., 2008; Li et al., 2012), arguing against the peripheral effects of the drug. Inhibitors more specific to NOS1 have also shown sensitivity to behavioral performance in spatial reference and working memory (Holscher et al., 1996; Zou et al., 1998; Yildiz Akar et al., 2009). Furthermore, NOS1 knockout mice show impaired spatial memory, social interactions and contextual fear memory (Weitzdoerfer et al., 2004; Kelley et al., 2009; Tanda et al., 2009). In contrast, NOS3 knockout mice exhibit enhanced spatial learning, retention and reversal learning in the Morris water maze but increased anxiety-like behaviors in the plus maze and the open arena (Frisch et al., 2000). However, spatial learning is comparable to controls in the radial arm maze (Dere et al., 2001), suggesting that NOS3 knockout confers a specific deficit in spatial learning and may therefore play a particular role in hippocampal plasticity, where it has been shown to play a role in LTP in concert with NOS1 (Hopper and Garthwaite, 2006; Phillips et al., 2008).

A simpler form of experience-dependent plasticity that can be quantified by measuring neuronal responses rather than behavior is the plasticity that results from whisker deprivation in the barrel cortex. Depriving a single whisker for several days leads to expansion of the area of cortex dominated by that whisker (Fox, 1992; Wallace and Fox, 1999). NO is implicated in the potentiation component of this plasticity as αNOS1 knockouts exhibited reduced single whisker potentiation (Dachtler et al., 2011). In parallel with the LTP studies (Hardingham and Fox, 2006), experience-dependent potentiation was only abolished in double knockouts of αNOS1 and GluA1 (Dachtler et al., 2011). Plasticity was present in double knockouts of NOS3 and GluA1, suggesting that αNOS1 is the important isoform in the cortex, probably due to the close association between αNOS1 and the NMDA receptor (see section The Cellular Location of NOS1). In further support of this idea, NMDA-dependent release of NO is impaired in αNOS1 but not NOS3 knockout mice (Dachtler et al., 2011).

Further analysis of plasticity in αNOS1 knockout mice reveals both LTP and experience-dependent potentiation are abolished in male but not female mice (Dachtler et al., 2012). This could either mean that male mice rely solely on NO-dependent forms of potentiation, or that some form of compensation for the lack of NOS1 takes place in the female knockout mice that does not occur in the males. The sex difference was not seen in wild-type animals suggesting that the latter is a possible explanation. The sex difference in the αNOS1 knockout mice may be of importance to interpreting stroke data because factors involved in LTP are often also involved in excitotoxicity. NOS1 has long been known to be a factor in ischemic damage in stroke (Huang et al., 1994), most likely through the association of αNOS1 and PSD-95 (Cao et al., 2005). However, the magnitude of ischemic damage differs depending upon sex. Male αNOS1 knockout mice have less ischemic damage than wild-types, while female αNOS1 knockout mice have more damage than their wild-type counterparts (McCullough et al., 2005).

### NO and plasticity at gabaergic synapses

Because NO can play a role in pre-synaptic plasticity, it also means that it is not restricted to act on a particular set of post-synaptic receptors or the protein trafficking machinery associated with them. Instead, in so far as the vesicular release machinery is common across transmitter systems, NO can potentially regulate release for several different neurotransmitters including GABA (Table 2).

**Table 2:** **The role of Nitric oxide in GABAergic function**.

**References**	**Title**	**Presynaptic action?**	**Retrograde messenger?**	**Effect?**	**Structure (preparation)**
Lange et al., 2012	Heterosynaptic long-term potentiation at interneuron-principal neuron synapses in the amygdala requires nitric oxide signaling	✓	✓	Effect on plasticity	Amygdala (slice)
Moreno-Lopez et al., 2002	Nitric oxide facilitates GABAergic neurotransmission in the cat oculomotor system: a physiological mechanism in eye movement control	✓	✓	Controls velocity responsiveness of PH neurons	Medial vestibular nucleus projection to prepositus hyperglossi (PH) neurons (*in vivo*)
Szabadits et al., 2007	Hippocampal GABAergic synapses possess the molecular machinery for retrograde nitric oxide signaling	✓	✓	Anatomical evidence: nNOS is post and sCG presynaptic	Hippocampus (*in vivo*)
Xue et al., 2011	NMDA receptor activation enhances inhibitory GABAergic transmission onto hippocampal pyramidal neurons via presynaptic and postsynaptic mechanisms	✓	✓	Increase in sIPSP frequency and amplitude	Hippocampal (slice)
Yang et al., 2007	Kv1.1/1.2 channels are downstream effectors of nitric oxide on synaptic GABA release to preautonomic neurons in the paraventricular nucleus	✓	✓	Nitric oxide acts on GABA via Kv1.1/1.2	Paraventricular nucleus of the hypothalamus (slices)
Yang et al., 2007	Kv1.1/1.2 channels are downstream effectors of nitric oxide on synaptic GABA release to preautonomic neurons in the paraventricular nucleus	✓	✓	Increases frequency but not amplitude of inhibitory minis	Paraventricular nucleus of the hypothalamus (slices)
Bright and Brickley, 2008	Acting locally but sensing globally: impact of GABAergic synaptic plasticity on phasic and tonic inhibition in the thalamus	✓	✓	Increases frequency of sIPSCs	Thalamus (slices)
Wall, 2003	Endogenous nitric oxide modulates GABAergic transmission to granule cells in adult rat cerebellum	✓	✓	NO modulates toninc GABA release	Cerebellum (slices)
Holmgren and Zilberter, 2001	Coincident spiking activity induces long-term changes in inhibition of neocortical pyramidal cells			Analogous to cases where nitric oxide is involved	Neocortical (slices)

Anatomical evidence implicates NO in regulation of pre-synaptic GABA release. In excitatory pyramidal cells in the hippocampus, NOS1 lies post synaptic to GABAergic synapses and the “NO receptor” (sGC) lies in the pre-synaptic terminals of those same GABAergic synapses, thereby providing both elements required for retrograde synaptic signaling in close assembly (Szabadits et al., 2007). In this case, rather than being associated with PSD95, which does not appear to localize at post-synaptic densities of symmetric synapses, GRIP1 may bind NOS1 at the post-synaptic site. The pre-synaptic terminals in question belong to parvalbumin- and CCK-containing cells that synapse onto somata and proximal dendrites of pyramidal cells. Consistent with this location, application of NO donors increases cGMP levels in GABAergic interneurons (Bartus et al., 2013). It is not clear how the endogenous signal arises to activate NOS1 at these inhibitory synapses, but one possibility is that action potentials could raise intracellular calcium via voltage gated calcium channels and the spatial localization of NOS1 immediately post-synaptic to the GABAergic terminals targets NO to the inhibitory terminals.

Physiological evidence further implicates NO in GABAergic synaptic plasticity. Evidence comes from observations on paraventricular neurons (Li et al., 2002), the prepositus hypoglossal nucleus (Moreno-Lopez et al., 2002), the amygdala (Lange et al., 2012) and thalamic projection neurons (Bright and Brickley, 2008). In the hippocampus, NMDA receptor activation in pyramidal cells causes an increase in spontaneous GABA_*A*_ receptor mediated IPSCs that are sensitive to an NO scavenger (Xue et al., 2011). In the ventral tegmental area, GABAergic synapses onto dopaminergic neurons express a pre-synaptic form of LTP that is dependent upon NMDA receptor activation, NO, GC, and PKG for its induction and maintenance and is selective to GABA_*A*_ synapses (Nugent et al., 2007, 2009). Furthermore, pre-synaptic GABAergic LTP from the lateral amygdala to the basolateral amygdala depends upon NO generated from glutamatergic neurons (Lange et al., 2012).

**Figure 7:**
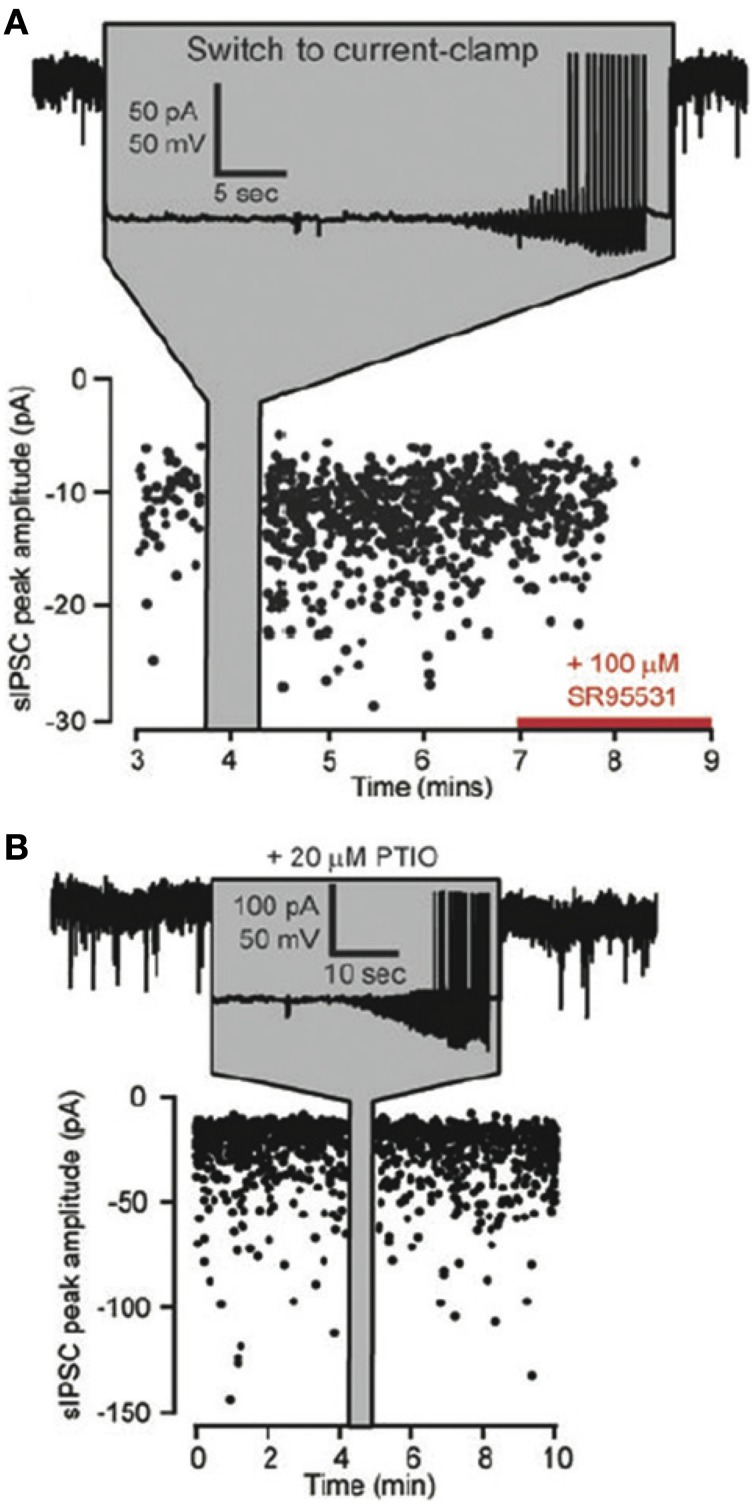
**Effects of NO on the frequency of GABAergic spontaneous IPSCs (sIPSCs). (A)** Plot of the peak amplitudes of spontaneous IPSCs against time for a thalamocortical (TC) lateral geniculate neuron. At the time indicated by the gray panel a switch is made from voltage to current clamp so that somatic action potentials can be generated. Note the increase in sIPSCs on returning to voltage clamp and the block of all sIPSCs by SR95531 toward the end of the experiment. **(B)** A similar recording from a TC relay neuron with the same protocol as in **(A)**, but in the presence of the NO scavenger PTIO (20 μ M) in the external solution. Note that GABAergic synaptic plasticity is blocked. Adapted from Bright and Brickley (2008) with kind permission of the authors and the Physiological Society.

The studies on GABAergic potentiation in the thalamus are particularly interesting because it only requires post-synaptic action potentials, which cause an increase in spontaneous GABAergic mIPSC frequency. This effect is blocked by the NO scavenger PTIO (Bright and Brickley, 2008) suggesting that the action potentials lead to release of NO that in turn produces changes in GABA release (Figure 7). The NO donor SNAP can also increase GABA mini frequency in these cells (Bright and Brickley, 2008). The sufficiency of post-synaptic action potentials in this study is reminiscent of the findings of Volgushev and colleagues in the visual cortex, who showed that post-synaptic action potentials produced NO-dependent potentiation in pyramidal cells (Volgushev et al., 2000) and Phillips et al. who showed that NO-dependent LTP in the hippocampus relies on somatic post-synaptic action potentials (Phillips et al., 2008). These findings raise the possibility that post-synaptic action potentials may simultaneously produce NO-dependent plasticity at inhibitory synapses and spike timing-dependent plasticity at excitatory synapses on the same cell, a property that may be involved in maintenance of inhibitory-excitatory balance.

### NO and the regulation of excitatory/inhibitory balance

Cells in the cortex exhibit a balance between excitation and inhibition such the ratio between inhibitory and excitatory conductances is relatively constant for different inputs. For layer 5 pyramidal cells in the visual cortex, the ratio of excitatory to inhibitory conductance has been estimated at 20:80 (Le Roux et al., 2006) using the method of Monier et al. (2008). The excitatory/inhibitory (E/I) balance would be expected to be a universal phenomenon as loss of E/I balance in favor of excitation leads to epilepsy. Consistent with this idea, inhibition has been shown to be matched to excitation in the visual (Anderson et al., 2000; Priebe and Ferster, 2005) auditory (Wehr and Zador, 2003, 2005) and somatosensory cortices (Wilent and Contreras, 2005). Studies have shown that NO may play a role in the maintenance of the E/I balance in the visual cortex. It can be demonstrated that the E/I balance is maintained in layer 5 pyramidal cells following potentiation by theta burst stimulation of cortical layers 2/3, 4 or 6 (Le Roux et al., 2006). Stimulating layer 4 and increasing endogenous levels of NO by dosing a cortical slice with L-arginine, or administration of an NO donor, also increases excitatory and inhibitory conductances in balance (Le Roux et al., 2009). These studies suggests that NO may play a homeostatic role in maintaining the balance between excitation and inhibition in the cortex.

## Conclusions

Deciphering the role of NO in the brain has not been a simple matter and at times the results of different studies have been confusing. Nevertheless, a clearer picture is now emerging of how NO might act to regulate synaptic function in the brain. In excitatory cells, NOS1 is located discretely in spines and is tethered to the post-synaptic membrane by its interaction with PSD95 in complete contrast to its location in a subpopulation of NOS1^+^ NPY^+^ inhibitory cells, where NOS1 is located in the cytoplasm along axons and dendrites and appears to be expressed at higher levels. The low levels of NOS1 expression in excitatory cells of the cortex and hippocampus dictate that under normal physiological conditions low concentrations of NO are evolved during stimulation by calcium, which in turn means that it has a relatively small range and is therefore probably synapse-specific in its action. The only obvious receptor that is sensitive at the low nM to pM range is guanylate cyclase, although there is some evidence for endogenous levels of proteins with nitrosothiol groups that would require higher concentrations of NO. There is a substantial body of literature that suggests that NO acts in a retrograde manner on several aspects of vesicular release and recycling, so much so that it would seem perverse to argue that NO does not act pre-synaptically at this point. Present evidence suggests that NO acts in a retrograde manner to affect not only glutamatergic synapses but also GABAergic synapses as well as other transmitter systems. Finally, there is substantial evidence in the literature that the retrograde route of action is important for plasticity in the cortex and hippocampus in both inhibitory and excitatory cells.

Nevertheless, a number of important questions remain about the action of NO at synapses. Two questions relate to the concentration of NO in the brain. First, are the levels of NO required for nitrosothiol production at the SNARE complex proteins actually achieved *in vivo*? Second, could higher levels of NO reported in some studies be generated by the higher NOS levels present in the NOS1^+^ GABAergic cells? A further set of questions relate to the action of NO at pre-synaptic GABAergic synapses. There is evidence that sGC is present in GABAergic terminals and that NOS1 lies post-synaptic to it (Szabadits et al., 2007). There is evidence that GABAergic mini EPSC frequency increases following somatic spiking in the LGn (Bright and Brickley, 2008). Therefore, what is the mechanism of post-synaptic spike-dependent potentiation of GABAergic transmission and is it indeed NO-dependent in the cortex and hippocampus? More generally, is this mechanism related to the post-synaptic spike potentiation present at excitatory synapses (Volgushev et al., 2000)? Unraveling this effect could help us understand whether the E/I balance is maintained by NO acting simultaneously on GABAergic and excitatory transmission (Le Roux et al., 2009). Finally, while we have concentrated on the pre-synaptic role of NO in this review, there is evidence that NO also has a post-synaptic action. In addition to activation of post-synaptic sGC, there is evidence that post-synaptic proteins have nitrosothiol groups, particularly those close to its PSD location (see Figure 1). If NO also has a post-synaptic role in plasticity it raises the additional question about whether it can play a homeostatic role in balancing or matching pre- and post-synaptic function. With a little good fortune, it will not take another 25 years of research to solve these and other related questions on the role NO plays in synaptic function in the brain.

### Conflict of interest statement

The authors declare that the research was conducted in the absence of any commercial or financial relationships that could be construed as a potential conflict of interest.
